# Evidence Based Dyadic Therapies for 0- to 5-Year-Old Children With Emotional and Behavioral Difficulties

**DOI:** 10.3389/fpsyt.2019.00677

**Published:** 2019-09-18

**Authors:** Reem M. A. Shafi, Ewa D. Bieber, Julia Shekunov, Paul E. Croarkin, Magdalena Romanowicz

**Affiliations:** Department of Psychiatry and Psychology, Mayo Clinic, Rochester, MN, United States

**Keywords:** young child, Psychotherapy, emotional regulation, behavioral issues, mental health

## Abstract

As many as one in four preschool-aged children are estimated to struggle with psychosocial stress and social-emotional issues; yet, interventions are often postponed until older ages when change is actually more difficult. Reasons for this include limited interventions, paucity of FDA approved medications for young children, as well as the dearth of clinicians adequately trained in psychotherapeutic approaches for young children. This commentary outlines indications of the four most commonly used evidence-based dyadic psychotherapies for young children: Child-Parent Psychotherapy (CPP) and Trauma-Focused Cognitive Behavioral Therapy (TF-CBT), used primarily for young children with trauma, and Parent-Child Interaction Therapy (PCIT) and Child Parent Relationship Therapy (CPRT), used mostly for children with behavioral issues. Rooted in attachment theory and further supported by the premise that the quality of the child–caregiver dyad is paramount to psychological wellbeing, these therapies focus on strengthening this relationship. Literature indicates that insecure or disorganized early attachments adversely affect an individual’s lifelong trajectory. These therapies have demonstrated efficacy leading to positive behavioral changes and improved parent–child interactions. The major challenges of clinical practice focused on young children and their families include proper diagnosis and determining the best therapeutic strategy, especially for families who have not benefited from prior interventions. At this time, it is still unclear which therapy is best indicated for which type of patients and it mostly has been driven by convenience and provider preference or training. Further research is required to tailor treatments more successfully to the child’s needs.

## Introduction

It is estimated that as many as one in four preschool-aged children struggle with psychosocial stress and social-emotional issues, both of which may be precursors to later disease (1). The period of 0–3 years is the most formative in human brain development (due to the critical period of synaptogenesis, pruning, and myelination) and carries the potential for lifelong consequences, both positive and negative (2, 3). However, despite the acknowledgement of increasing pediatric mental health concerns and interventions aimed at reducing the raising suicide rates, the quality of training, implementation, and dissemination of interventions focused on the 0- to 5-year-old patient population remains inadequate.

While many psychiatric disorders emerge in adolescence (2), their underpinnings can be seen much earlier. For example, oppositional defiant disorder (ODD) is a highly prevalent condition in young children (6–15%) (3). Yet, the ones who develop serious externalizing behaviors early in life have a two- to three-fold higher risk of developing conduct disorder (CD), which is more difficult to manage and has a worse prognosis (4–6). According to a longitudinal follow-up study by Luby et al. (2009), internalizing disorders such as major depressive disorder (MDD) in preschool children also show stability over time. Here, preschoolers diagnosed with depression at baseline had a higher risk of developing depression at 12 and/or 24 months later in comparison to the control group (7). In another study by Luby et al. (2014), preschool-onset depression was shown to be a predictor of MDD in later childhood. In addition to non-supportive parenting, the Adverse Childhood Experiences (ACE) score could also affect depression severity and oppositional behaviors (8–10).

Importantly, early interventions aimed at emotional and behavioral disturbances are more effective when done at preschool age rather than school age (11). Since early childhood onset of externalizing disorders (such as ODD or CD) and internalizing disorders (such as MDD or anxiety disorder) can have a lasting, detrimental impact on later life, it is imperative that early identification by trained providers occurs with implementation of safe, effective psychotherapeutic treatment modalities (11).

## Psychotherapy With Young Children—General Overview

Infant and Early Childhood Mental Health has been recognized by the field of psychiatry for over 75 years. There are a number of clinical interventions driven by various theories; however, it is safe to say that a great majority of them are founded on dyadic psychotherapy. Young children do not function without their caregivers, and as such, dyadic therapies aim to effectively address the parent, the child, and their relationship. The latter is particularly important as young children function and grow only within the context of their relationship with caregivers.

Most dyadic therapies try to address at least three different “parties” involved. It is a difficult task and attrition rate is usually high. Zeanah and Boris (12) describe several challenges of dyadic treatment. First, young children develop and change rapidly between 0 and 5 years old. This rapid development necessitates an urgency to address any issues so that ongoing development continues and acquisition of milestones is reached appropriately. Children have a limited repertoire of self-expression for some time and often irritability or anger can be a sign of a number of diagnostic categories which further complicates assessment. Clinicians have to rely predominantly on parental or other caregiver descriptions of the child’s behaviors, which may be inaccurate. Lastly, while the debate as to whether nature or nurture contributes to human behavior has largely subsided with acknowledgment of the contribution of both, including the interactions with the caregiver, it remains challenging in a clinical setting to precisely identify which of the factors plays the most pivotal role for a particular patient.

There are also a number of external factors that limit access to care for young children and their families for early symptoms. Unfortunately, even with optimal identification of children who could benefit from psychiatric or psychotherapeutic treatment, there is a critical shortage of qualified providers, particularly in infant mental health. Unfortunately, despite numerous studies, there remains a misconception among some providers that young children will grow out of their difficulties or simply forget traumatic experiences (13). Clinicians working with these children need to be attuned to this and be familiar with both child and adult psychopathology, which requires specialized training.

This commentary outlines indications of the most commonly used evidence-based dyadic psychotherapies for young children: Child-Parent Psychotherapy (CPP), Trauma-Focused Cognitive Behavioral Therapy (TF-CBT), Parent-Child Interaction Therapy (PCIT), and Child Parent Relationship Therapy (CPRT). CPP, TF-CBT and CPRT are used primarily for young children with trauma, and (PCIT) is used mostly for children with behavioral issues.

All four therapies are well-known. The basic theory describing their background and detailed evidence of efficacy is beyond the scope of this article. Here, we will summarize the primary characteristics of each treatment’s methods, paying special attention to the indications, goals, and specific interventions.

## Child-Parent Psychotherapy (CPP)

CPP was initially studied as a treatment for traumatized children and their parents and continues to be the main indication. However, it has since been found to be useful for early childhood disorders such as anxiety, bonding difficulties, behavioral issues, and others. The main premise of CPP, recommended for children ages 0–6, is to attempt to help the child’s mental health issues while supporting and strengthening primary attachment relationships (14). The attachment system between the child and caregiver is the leading organizing system that helps the child with his or her responses to dangerous situations. A trusting relationship within this paradigm is the most important protective factor for a young child (14, 15).

A manual of CPP, “Don’t Hit My Mommy” (16), is available, but master level clinicians are required to complete formal training and supervision in order to be considered qualified providers. CPP takes approximately 1 year to complete and consists of the therapist meeting with the child and parent on a weekly basis. The initial assessments are typically scheduled with the primary caregiver to create the treatment plan; they are then followed by joint child–parent play sessions. The therapist selects developmentally appropriate toys before each session for the child to use. If there is a history of trauma, toys usually are used to elicit trauma material. During the course of the treatment, the therapist may schedule individual sessions with the primary caregiver as clinically indicated to review progress.

CPP is a psychodynamic oriented treatment, and interpretation during play is one of the main techniques. In order to address both the parent and the child, the therapist delivers their interpretation in a two-prong way. The main goal of the interpretation is to show compassion for the parent (many of whom have experienced past abuse or traumatic events) and help them realize, in the presence with their child, that their previous experiences affect their relationship. The idea behind such an approach is that the better parents remember their past, the less likely it is for them to repeat it with their children. It is also easier for parents to be more protective and have compassion modeled by the therapist for their child. The danger of burying traumatic events and not being able to talk about them is despite best intentions there is the potential of parental reenactment of maladaptive events with their own children. Parents may also use defense mechanisms such as denial or avoidance to fend off uncomfortable feelings, impacting their ability to enjoy the relationship with their child (17).

In CPP therapy, the therapist is required to be fairly flexible and creative when working with patients. The length of sessions often depends on the level of engagement of the child and parent. Depending on age, this may vary from 30 to 45 min. Infants always accompany their parents during sessions, but with toddlers and preschoolers, the session may be divided. This may involve spending a portion of the session with the child and in the remainder the therapist engages in “grown up talk.” Alternatively, the therapist may meet separately with the parent and then the child (especially in situations when parental mental health is in peril and needs to be addressed immediately).

The main goals of CPP may be divided into two categories: global goals and trauma-related goals. Global goals of CPP revolve around supporting normal development and helping the child and parent develop a strong and loving relationship. Part of this includes teaching the parent how to help the child manage their emotions and control their behaviors. Trauma related goals are directed to help both the child and parent resolve trauma related symptomatology, re-build trust, and normalize their responses. Through play and the therapist’s interpretations, the child and parent are encouraged to create a trauma narrative that is meaningful to them both and developmentally appropriate to the child (18).

CPP was empirically validated in a randomized controlled study conducted by Lieberman et al. on a sample of 75 preschoolers who experienced domestic violence (19). Since then, CPP has been well studied in at least nine randomized controlled trials with sample sizes ranging from 50 to 198 dyads across a number of different populations, including but not limited to Latino toddlers and their mothers with a history of trauma (20), depressed mothers and their toddlers (21), and children from the welfare system with a history of abuse (19). The effect size in the seminal paper by Lieberman et al. was around 0.4 both for the child and the parent (22).

## Trauma Focused Cognitive Behavioral Therapy (TF-CBT)

One of the most common evidence-based treatments for trauma in children is TF-CBT (23, 24), a treatment model delivered to young children that is a components-based treatment, requiring full involvement of the child’s non-offending caregivers. Although initially tested on children with a history of sexual abuse, it has since been successfully applied to children, ages 3–18, with diverse and sometimes complex trauma: physical, sexual, and emotional or neglect. TF-CBT also addresses parental secondary trauma and helps parents with addressing difficult behaviors in their children that resulted of trauma. Typical treatment involves 8–20 sessions divided between individual sessions for the child and the caregiver, followed by conjoint sessions. Due to children’s short attention spans, especially for exploring difficult trauma, sessions should not exceed 30 min in length. The remainder of the session can be devoted to the caregiver, or brief activities such as learning relaxation skills, coloring, or other positive activities that allow the child to take a short break.

TF-CBT is structured so that the child and caregiver both gradually engage in exposure exercises. This begins at the assessment session. First, the therapist must ensure that the child has memory of the trauma and sufficient verbal skills to acknowledge it took place. Then slowly, the child and caregiver participate in the process of learning coping skills, such as relaxation skills and deep breathing, to help manage reminders of the trauma. They are also encouraged to narrate the trauma story and make meaning of their experience.

Engaging primary caregivers from the very beginning is crucial for a number of reasons, starting with compliance. Caregivers who understand the indications and goals of therapy are more likely to complete treatment (25, 26). Additionally, the primary focus is on positive parenting skills, largely praising, and rewarding good behaviors. Parents are taught that negative consequences may be used but that relying on them without praise is not sufficient to change behaviors. Negative consequences are intended to be benign and brief (for example a short time-out). Psychoeducation is provided to both the child and their caregivers. child and their caregivers. Caregivers are counseled on the differentiation between problematic behaviors that are in the context of trauma and normal behaviors that are to be expected as a normal course of development. This should help the caregivers avoid misinterpreting new behaviors as pathological in the aftermath of trauma and to respond effectively (23, 27).

Psychoeducation to children should be delivered in a developmentally appropriate way and the therapist is encouraged to assess the child’s understanding of the material as the treatment progresses. The relaxation and affect expression training are very similar to other CBT treatment protocols in that even very young children are capable of discussing simple emotions such as sad, mad, and happy. Likewise, young children can be taught cognitive coping skills, for example, with the help of age-appropriate books (28). Additionally, one of the most important elements of treatment is trauma narrative, which can be accomplished by a creation of a book with drawings and stories of trauma. If the child is very young and prefers to play, then dolls, puppets, and other play materials may be used. Narration of trauma provides an opportunity to plan for *in vivo* mastery. Often planning for gradual exposures with caregivers is necessary, as avoidance behaviors frequently occur outside of therapy sessions. It is very helpful to share the trauma narrative in conjoint sessions towards the end of the treatment. Conjoint sessions are also beneficial in practicing communication between the caregiver and the child, and to identify behavioral interventions that are best for the dyad.

TF-CBT is manualized and certification typically consists of formal in-person training as well as consultation calls (29, 23). The most commonly cited study supporting the use of TF-CBT in young children by Cohen et al. (30) found TF-CBT to be superior to nondirective supportive therapy in addressing sexual trauma. Specifically, there was a significant decrease in PTSD symptomatology and sexualized behaviors in TF-CBT subjects even at 1 year follow up in comparison to the control group. Since then, further studies have found TF-CBT to be beneficial in addressing symptoms resulting from emotional and physical abuse, natural disasters, and complicated grief (24, 25, 31). There are at least 10 randomized control studies with sample sizes ranging from 36 to 229 dyads and 6 review studies of TF-CBT. Studies showed medium to large effects that were defined as standardized mean differences of Cohen’s d ≥.40 (medium effects), and large effects were defined as d ≥.75 (32).

## Parent Child Interaction Therapy (PCIT)

PCIT is an evidence-based behavioral treatment for 2–7 year olds with behavioral disturbances arising from internalizing and externalizing disorders (33). The aim of treatment is to improve symptoms by improving the child–caregiver relationship. The distinguishing feature of PCIT is the use of a “bug in the ear system” that allows the therapist to coach the caregiver in real time. This discrete method allows for in the moment training and feedback as the caregiver interacts with their child while the therapist watches and teaches behind a one-way mirror.

PCIT is implemented in two stages: Child Directed Interaction (CDI) and Parent Directed Interaction (PDI). CDI involves teaching PRIDE (Praise, Reflection, Imitation, Description, and Enthusiasm) “do” skills that the caregiver is taught to utilize during interactions and play with the child (**Table 1**). At the same time, the caregiver is taught to avoid “don’t” skills during CDI (**Table 2**). Once mastery of the PRIDE skills is attained, the therapy transitions onto PDI. Here, the caregiver learns appropriate disciplinary techniques, such as effective time-outs, and the phrasing of clear directions or commands (direct commands) (34, 35). Sessions are usually 45 min long, although in the second part of treatment- PDI, they may be extended to 60–90 min depending on the child’s response. For example, if the child does not comply with a direct command given to them by their parent, they have to sit in a time out chair for 3 min followed by 5 seconds of silence (the latter is important for the child to learn that only calm behavior will get them out of the chair). Likewise, if the child gets off the chair prematurely, they have to go to the time out room, or if the room is unavailable, parents practice a “swoop and go” strategy, where they collect all the toys that are in the room and tell the child that they will be standing right outside the door until the child is ready to sit in the chair. The recommendation is that parents should wait at least a minute that is followed by 5 s of silence before approaching the child to put them in the time out chair. PDI also includes a public outing session where parents have the opportunity to practice their newly acquired skills in a park or a store. There is also a sibling session where parents practice their skills with all their children. This is particularly helpful for children who have difficult relationships with their siblings.

**Table 1:** (19, 34, 35) CDI “do” skills.

CDI “do” skills	Explanation	Example
Praise	Caregiver is encouraged to praise all good behaviors in order to positively reinforce them and increase their frequency. Of the two types (Labeled and Unlabeled Praise), labeled praise is preferred as it clearly specifies the behavior for which the child receives praise.	Labelled Praise: “I love how you are sitting quietly”, “Thank you for using your walking feet” Unlabeled Praise: “Great Job!”, “Nice work!”
Reflection	Caregiver repeats or summarizes what child says, showing them they are attentive to them. Additionally, this helps to increase the child’s vocabulary.	Child: “I like apples” Caregiver: “You like apples” Child: “I don’t know how to fix this truck” Caregiver: You are wondering how to fix the truck”
Imitation	Caregiver allows the child to lead play and imitates positive behaviors.	Child: Begins to draw a sun Caregiver: Also starts drawing a sun
Behavioral Description	Caregiver acts as a “sports commentator” describing what the child is doing, creating an opportunity to teach without asking questions.	Child: Playing with a cube Caregiver: “You are picking up the yellow cube and putting it on top of the box”
Enjoyment	Caregiver expresses enthusiasm during the play and learns to enjoy the time with the child encouraging engagement.	Therapist: “(Child) loves spending time with you”, “When you smile it really causes (Child) to brighten up!”

**Table 2:** (19, 34, 35) CDI “don’t skills”.

CDI “don’t skills”	Explanation	Examples
Questions	These can push the adult’s agenda and inhibit child-lead play.	“What color is this?” “Where do you want to put this block?”
Commands	Indirect Command: often phrased as a question and offers a choice. Direct Command: clear and explicit in what parent expects of child.	Indirect: “Can you put your coat on?” Direct: “Put your coat on please”
Criticism	Caregivers are taught to avoid criticism of any kind.	“No, silly” “That is not a clever way to fix it!”

PCIT has a wide body of evidence supporting its use, including randomized controlled trials indicating long term improvement in parenting strategies and diminished behavioral issues, as compared with treatment-as-usual groups (36). PCIT has traditionally been used for developmentally normal children between the ages of 2–7 years. However, there is evidence that PCIT can be modified to benefit children with behavioral problems as a result of autism, severe developmental delay, and intellectual disability (37–39). In addition to externalizing conditions such as ODD, adaptations to PCIT have increasingly targeted and been found to be efficacious for internalizing conditions such as depression and anxiety.

Luby et al. (2018) developed PCIT with an Emotion Development (ED) component as an addition to the therapy. This is now referred to as PCIT-ED and lengthens the series from an average of 12 to 18 sessions. After completion of standard PCIT, the caregiver is taught specifically how to coach the child to identify and understand emotions in themselves and others with a stronger emphasis on the caregiver acting as an external regulator of the child’s emotions (2, 40). PCIT based treatments such as the Coaching Approach behavior and Leading by Modeling (CALM) Program have been effectively used to treat a spectrum of anxiety disorders by focusing on parent led exposures and behavioral modeling (41).

In summary, PCIT has applicability outside its traditional indications of normally developed children with externalizing disorders. Standard PCIT technique and adaptations described above offer safe and effective alternatives to psychotropic medications for a range of externalizing and internalizing disorders such as oppositional defiant behaviors, ADHD, anxiety, depression, and trauma in young children (2).

Based on comparative studies, PCIT demonstrated large effect sizes for helping with negative parent and child behaviors as well as increasing positive parenting skills and improving child behaviors (42). There are at least 12 meta-analyses and reviews of PCIT research (43, 44). Based on one analysis, PCIT has a large effect size of d = 1.65 (decrease in externalizing symptoms in children) (45). PCIT has also been studied in various settings such as in school therapy (TCIT- Teacher Child Interactive Therapy) (46), in-home settings (47), mothers who are incarcerated, as well as Primary Care Practice (group format) (48, 49).

## Child–Parent Relationship Therapy (CPRT)

CPRT is an evidence-based humanistic, child-centered, and play-based treatment program. It was initially developed for children ages 3–8 years old with more recent adaptations available for 1- to 3-year-old children as well as pre-teens. Other components of the treatment are 30 min, weekly, non-directive play sessions for the parent and the child (50, 51). Parents use skills learned in group therapy in their play interactions with children. Parents practice in a supervised environment how to better address their child’s emotional and behavioral needs and children learn that they can always depend on their parents. CPRT utilizes concepts of Child Centered Play Therapy (CCPT). Therapy is designed for a wide range of issues that include attachment disorders or difficulties bonding, for adoptive families and for those with a history of abuse in families with resulting emotional and behavioral problems in children. For the latter group, the aims of the treatment include decreasing parental stress and their secondary trauma symptoms, and helping with their child’s behavioral issues (52–55). Behavioral issues are addressed by parents serving as role models for their children and their ability to interact with them in a non-judgmental way with empathy and respect.

Group sessions in CPRT are structured and address various components that include education on CCPT skills (delivered mostly in sessions 1–3), as well as supervision and discussion of the video recordings of the in-home play sessions in a small group format (delivered mostly in weeks 4–10). Parents have the opportunity to process their concerns and ask appropriate questions as well as receive support (56).

Bratton et al. formalized the CPRT created treatment manual available for the therapists (56). The certification process contains a number of steps for mental health providers to undertake, including educational training, which allows therapists to conduct group therapy but not home visitations (parents may get certified as well). In addition, therapists must receive training in CCPT, complete CPRT certification exam, and have sufficient supervision of their clinical experience.

CPRT is considered an evidence-based treatment model based on at least 20 outcome studies that included 15 Randomized Control Trials (57). Based on a meta-analysis (Bratton et al., 2005) that assessed 93 studies, the effect size for CPRT is in the moderate range (d = 0.80). CPRT also has been implemented in various settings (e.g., school based) and across diverse populations (57, 58).

## Conclusions

Significant psychiatric conditions such as depression, anxiety, post-traumatic stress disorder, and ODD occur in very young children and early identification and treatment is of paramount importance. The long term effects of psychotropic medication remain uncertain, though its use has become increasingly common (2). The treatment modalities of CPP, TF-CBT, PCIT, and CRPT are evidence-based, valuable options for young children with a history of trauma, internalizing and externalizing disorders, and their caregivers. See **Table 3** for a summary of individual goals, indications, and length of treatment for each therapy. By focusing on the powerful influence of a positive caregiver–child relationship during a sensitive period of neurodevelopment these therapies may positively impact a child’s trajectory.

**Table 3:** Summary of three main evidence-based psychotherapeutic interventions in young children.

Type of therapy	Age (years)	Indications	Goals of therapy	Length of treatment/frequency and duration of sessions
CPP	0–6	PTSD and other trauma related disorders, anxiety, behavioral issues, attachment difficulties	Global Goals: Improve child–caregiver attachment Trauma Goals: Reduce trauma related symptoms	1 year/1–2 times a week/30–60 min
TF-CBT	3–18	PTSD and other trauma and stressor related disorders Grief	Gradual exposure to trauma narrative and learning of coping skills as a way to reduce symptoms	8–20 sessions/weekly/30 min
PCIT	2.5–7	Disruptive behavior in context of ODD, CD, autism, attention deficit hyperactivity disorder (ADHD), anxiety, selective mutism, depression	Increase positive interactions between caregiver and child, improve communication and teach appropriate disciplinary techniques to strengthen the caregiver–child relationship	8–12 sessions/weekly/45–90 min
CPRT	3–8	Attachment difficulties, children of adoption, families with history of abuse	CPRT utilizes concepts of Child Centered Play Therapy (CCPT). The main aim is to strengthen or create secure attachment between parent and a child. Behavioral issues are addressed by parents serving as role models for their children and their ability to interact with them in non-judgmental way with empathy and respect.	10 sessions of weekly group therapy (for parents only)/120 min plus 30 min weekly in home, video recorded play therapy (parent and child)

Although many providers are familiar with one of the discussed treatment modalities, it is very rare to find a clinician trained in multiple therapies. One of the major challenges in clinical practice with young children and their families is proper diagnosis but also being able to determine the best therapeutic strategy, especially for families who have had a weak or null response with previous treatment.

In TF-CBT, the non-offending caregiver is required to participate in the sessions that are different from CPP or CPRT where an abusive parent, as long as they are motivated for change, would be a viable candidate for treatment. This is an important aspect of various treatments that needs to be taken into consideration. Similar to PCIT and CPRT, parents who, for example, have lost custody due to abuse and who are now interested in reconciliation may use the treatment to reconnect with their children. Another important factor is the age of the patient. All four therapies are designed for very young children; however, only CPP takes on infants 0–12 months. TF-CBT also does not accept patients with dangerous acting-out or suicidal behaviors. While suicidal behaviors would be extremely rare in children 3–6 years old, dangerous acting-out is not uncommon, especially in patients with a history of significant abuse. It is often a difficult clinical decision whether to first address the behaviors with treatment such as PCIT or focus on the traumatic response with a referral to TF-CBT, CPP, or CPRT. Rarely is it a good idea to conduct both therapies at the same time due to “dilution” of the effectiveness of each therapy and high dropout rate. Although dual therapy is typically avoided, there remains a lack of guidance and no studies on the type of therapy that is best suited for a particular patient, and until recently, this was mostly driven by therapist’s availability and training as well as the patients’ preference.

In the absence of evidence and based on clinical practice alone, children who do present with such difficult behaviors are referred to PCIT for stabilization before undergoing TF-CBT, CPP, or CPRT to learn how to better cope with their trauma history. That being said, all four therapies address difficult behaviors in some capacity. CPRT is the only therapy that offers group sessions for parents, and if that is their preference, such therapy should be considered as a first line treatment. Further research is required to tailor the type of treatment quickly, more successfully, and according to the child’s and caregiver’s needs.

## Author Contributions

RS and MR drafted the manuscript. EB, JS, and PC made revisions to the manuscript. All authors read and approved the final manuscript.

## Conflict of Interest Statement

The authors declare that the research was conducted in the absence of any commercial or financial relationships that could be construed as a potential conflict of interest.
